# Publisher Correction: Enhanced β-adrenergic signalling underlies an age-dependent beneficial metabolic effect of PI3K p110α inactivation in adipose tissue

**DOI:** 10.1038/s41467-020-14939-0

**Published:** 2020-02-21

**Authors:** Caroline Araiz, Anqi Yan, Lucia Bettedi, Isabella Samuelson, Sam Virtue, Anne K. McGavigan, Christian Dani, Antonio Vidal-Puig, Lazaros C. Foukas

**Affiliations:** 1 Institute of Healthy Ageing & Department of Genetics, Evolution and Environment, University College London, London, WC1E 6BT UK; 2 University of Cambridge Metabolic Research Laboratories, Wellcome Trust-MRC Institute of Metabolic Science, Addenbrooke’s Hospital, Cambridge, CB2 0QQ UK; 3 Wellcome Trust Sanger Institute, Hinxton, CB10 1SA UK; 4 Université Côte d’Azur, CNRS, Inserm, iBV, Faculté de Médecine, 06107 Nice Cedex 2, France; 5 Present Address: National Institutes of Child Health and Human Development (NICHD), Bethesda, MD 20814 USA; 6 Present Address: University of Cambridge Metabolic Research Laboratories, Wellcome Trust-MRC Institute of Metabolic Science, Addenbrooke’s Hospital, Cambridge, CB2 0QQ UK

**Keywords:** Cell biology, Physiology

Correction to: *Nature Communications* 10.1038/s41467-019-09514-1, published online 4 April 2019.

The original version of this Article contained an error in Fig. 5a, in which the representative Western blot images for Akt phosphorylation in iWAT were a duplication of those for vWAT. This has been corrected in both the PDF and HTML versions of the Article.

