# Supplementary material for: Enhanced β-adrenergic signalling underlies an age-dependent beneficial metabolic effect of PI3K p110α inactivation in adipose tissue
Source: Nat Commun. 2019 Apr 4;10:1546. doi: 10.1038/s41467-019-09514-1 (PMC6449391; doi:10.1038/s41467-019-09514-1)

Supplementary Information

Enhanced β-adrenergic signalling underlies an age-dependent beneficial metabolic effect of PI3K p110α inactivation in adipose tissue

Araiz et al.

Contents:

-Supplementary Methods

-Supplementary Table 1

-Supplementary Figures 1-10

-Uncropped western blot images

Supplementary Methods

Blood spot extraction, glucose derivatisation and GC-MS analysis

Blood spots cards were extracted in 90% ethanol. Ethanol extracts were dried by speedvac and then underwent aldonitrile-pentaacetate derivatisation as follows: 50 µl of pyridine containing 2% hydroxylamine was added and samples were heated for 45 minutes at 90° C. Samples were allowed to cool and then 100 µl of acetic anhydride was added and samples were heated for 30 minutes at 60°C. Samples were cooled and then transferred to a heat block and dried at 60º C under nitrogen. Samples were then dissolved in 200 µl of ethyl acetate and spun down to remove crystalised hydroxylamine before the supernatant was transferred to an autosample vial. Mass spectrometry was performed using an Agilent 7890B GC and Agilent 5977A MSD using a 0.25 um x 30 m DB-5 (Cat: 122-5031, Agilent) column were used. GC conditions were as follows:

Injection and inlet: 1 µl of sample was injected at split 1:20.

Inlet temperature: 250ºC.

Oven Temperature: Step 1 80 °C for 1 min. Step 2 20ºC/min until 280ºC then hold for 3 minutes.

Column: 0.25 um x 30 m DB-5 (Cat: 122-5031, Agilent)

Flow rate: 1 ml /minute helium

Transfer heater: 290°C

Mass Spec: The mass spec was operated in scan mode between 45 and 850 D at 4 Hz. The source temperature was 230°C. Quad temperature was 150°C

The enrichment of 6,6-D2 D-glucose in blood was assessed by monitoring the 217-221 m/Z ions and calculated using multiple linear regression using the solver function of Excel.

Supplementary Table 1. List of q-PCR primers used in the present study.

**Primer name**	**Primer sequence (5'→ 3')**
Ucp1 forward	GGCCTCTACGACTCAGTCCA
Ucp1 reverse	TAAGCCGGCTGAGATCTTGT
Cidea forward	TGCTCTTCTGTATCGCCCAGT
Cidea reverse	GCCGTGTTAAGGAATCTGCTG
Pgc1a forward	AACCACACCCACAGGATCAGA
Pgc1a reverse	CTCTTCGCTTTATTGCTCCATGA
Dio2 forward	TGCGCTGTGTCTGGAACAG
Dio2 reverse	CTGGAATTGGGAGCATCTTCA
Elovl3 forward	AAGGTTGTTGAACTGGGAGACA
Elovl3 reverse	GTGGTGGTACCAGTGGACAAA
S18 forward	TCGAGGCCCTGTAATTGGAA
S18 reverse	CCCTCCAATGGATCCTCGTT

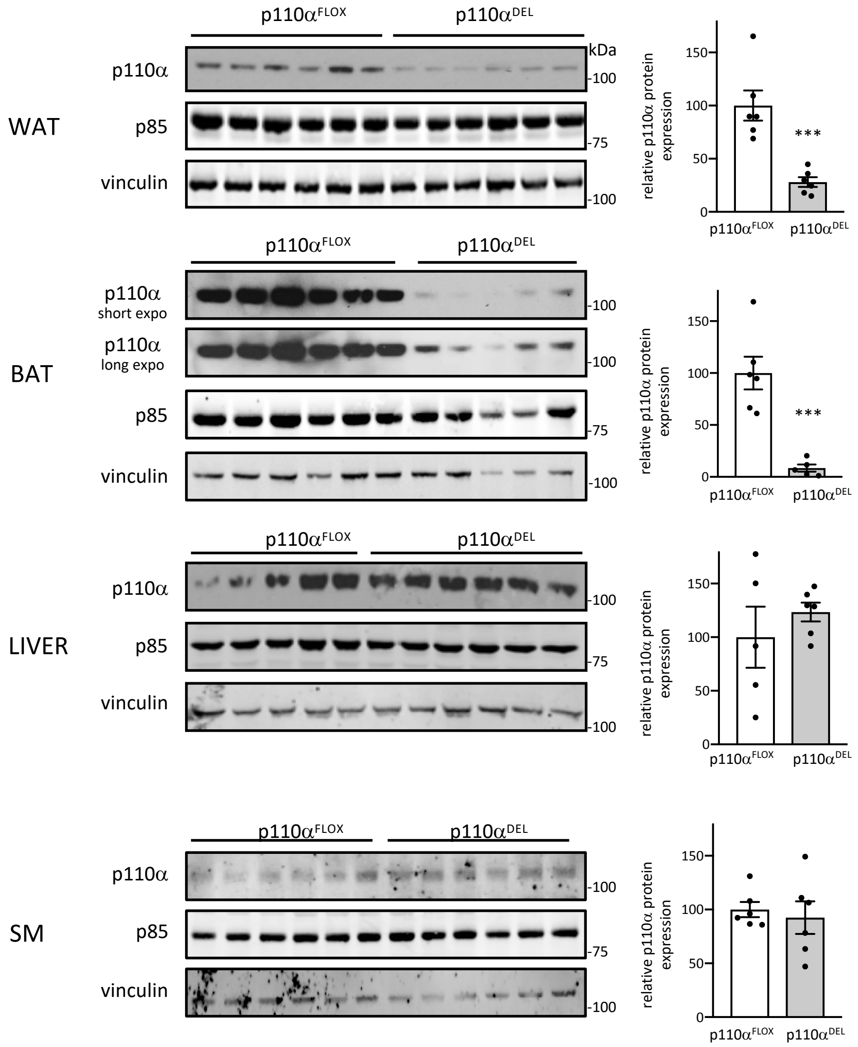

Supplementary Figure 1. p110α expression in metabolic tissues of p110α^{DEL} mice.
Expression of the p110α protein in white adipose tissue (WAT, epididymal), brown adipose tissue (BAT, interscapular), liver (L) and skeletal muscle (SM, gastrocnemius) of 1.5 year old p110α^{FLOX} and p110α^{DEL} male mice, assessed by immunoblot analysis. p110α was detected by enhanced chemiluminescence (ECL) and semi-quantitative data are presented in the respective graphs. Each lane represents a sample from an individual mouse (n=6 per genotype).
Data are presented as mean ± SEM. Statistical analysis by unpaired two-tailed t-test. *** $p<0.001$.

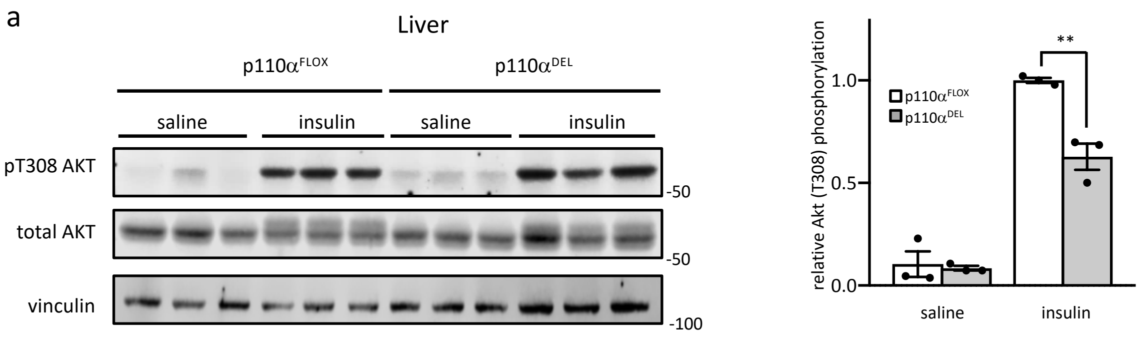

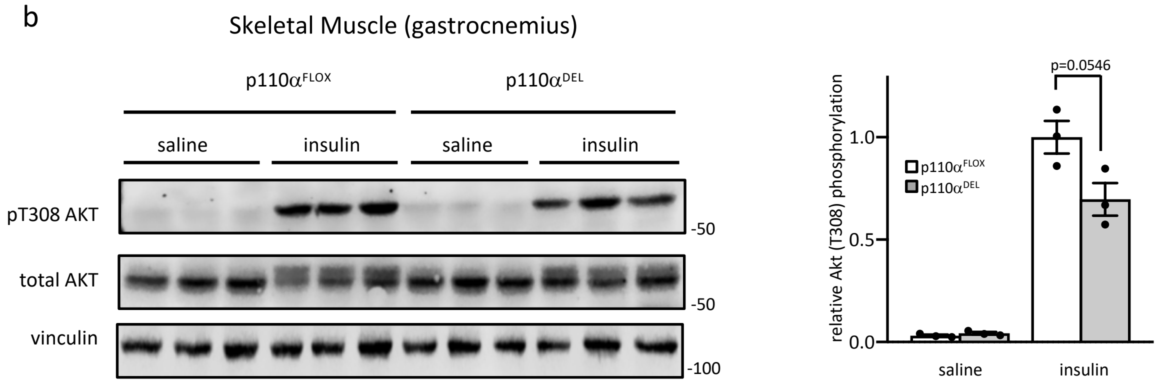

Supplementary Figure 2. Impaired insulin sensitivity in liver and skeletal muscle of p110α^{DEL} mice.

One year old mice (n=3 per genotype) were injected intraperitoneally with 100 mU g^{-1} of insulin. After 30 min, tissues were excised and snap frozen. Liver (**a**) and skeletal muscle (**b**) lysates were analysed for Akt T308 phosphorylation by immunoblotting. Vinculin was probed as loading control. Quantitative data of Akt T308 phosphorylation are shown in the respective graphs.
Data are presented as mean ± SEM. Statistical analysis by unpaired two-tailed t-test. ** p<0.01.

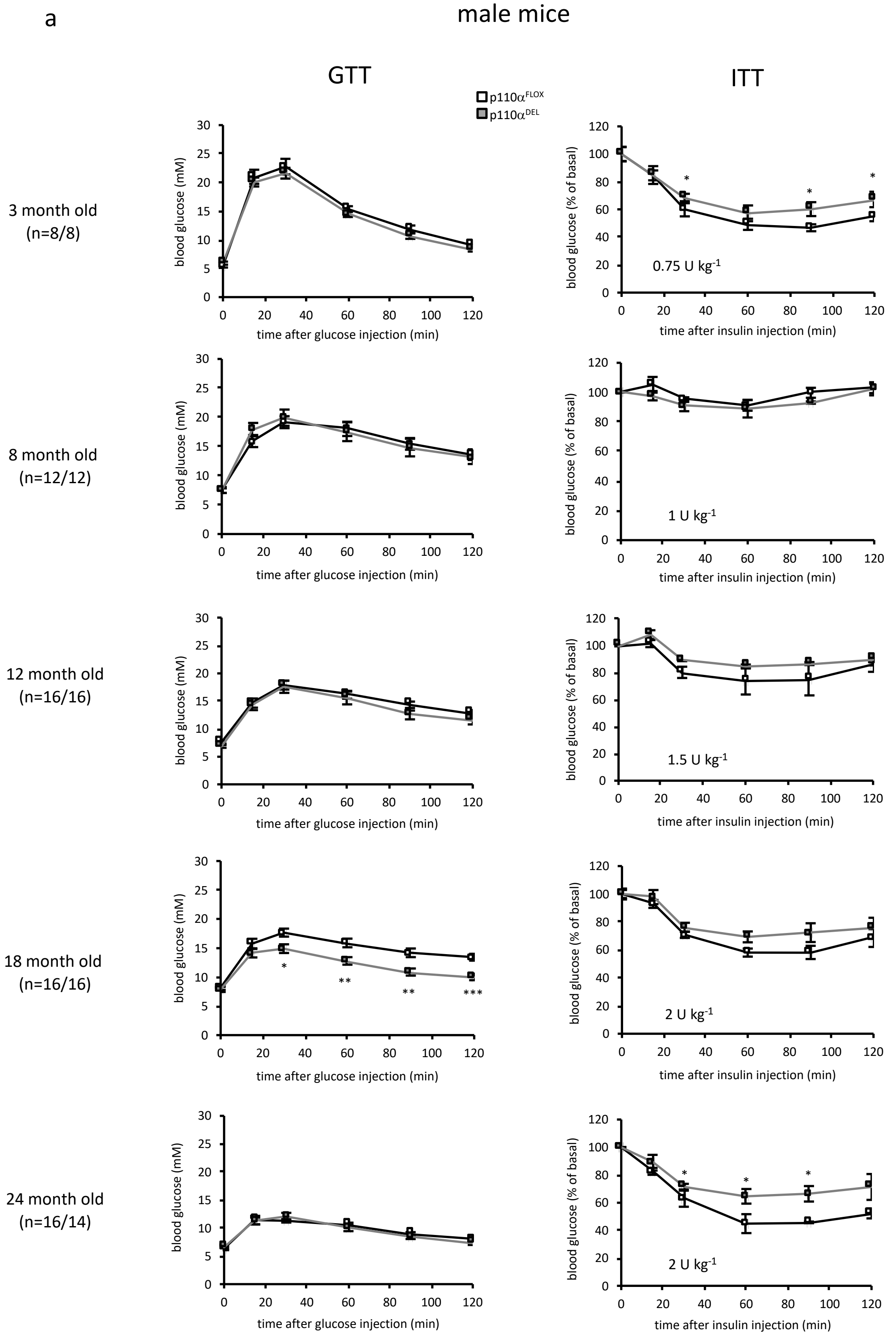
a
male mice
GTT
ITT
p110α FLOX
p110α DEL
3 month old (n=8/8)
8 month old (n=12/12)
12 month old (n=16/16)
18 month old (n=16/16)
24 month old (n=16/14)
blood glucose (mM)
time after glucose injection (min)
blood glucose (% of basal)
time after insulin injection (min)
0.75 U kg-1
1 U kg-1
1.5 U kg-1
2 U kg-1
2 U kg-1

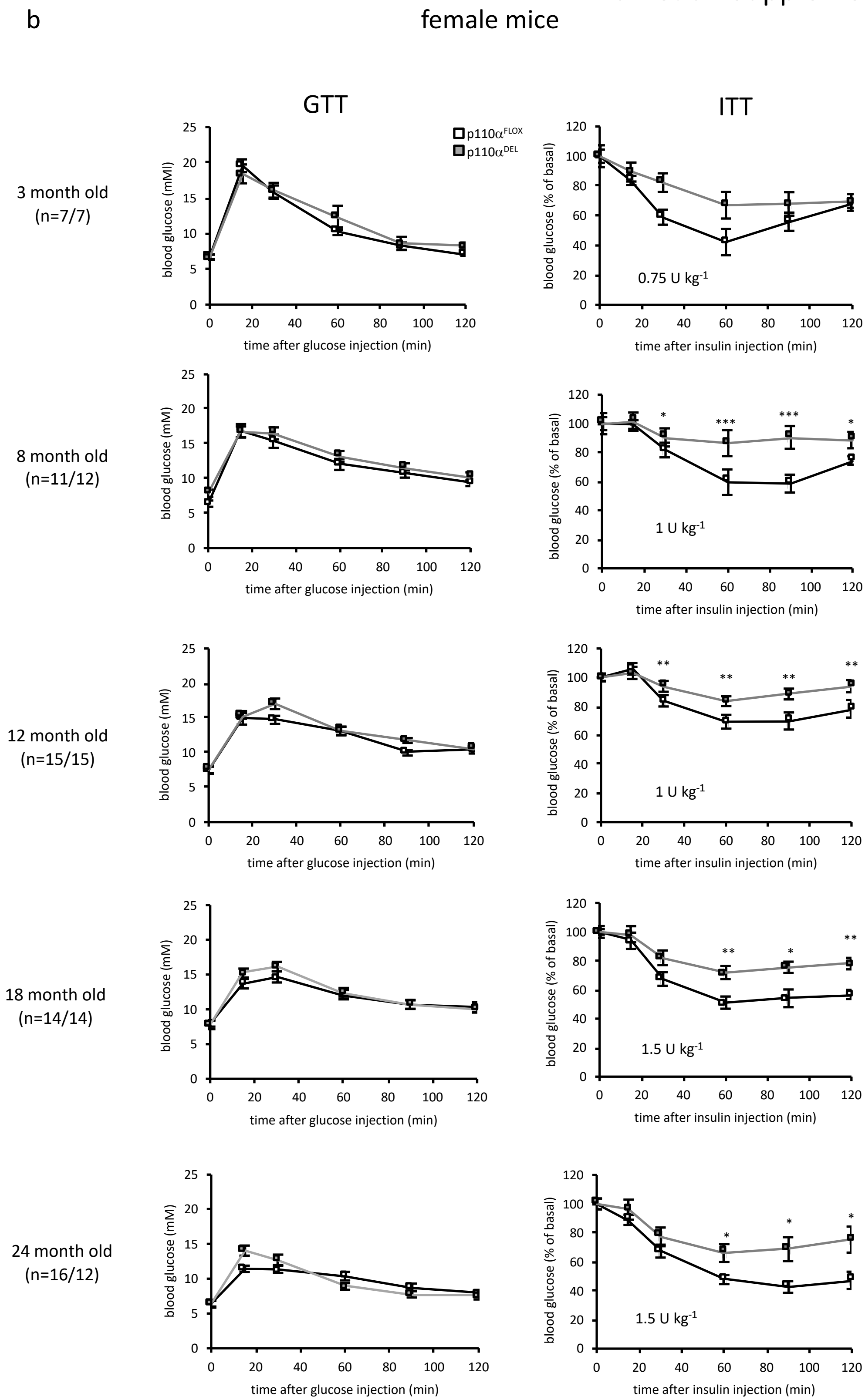

Supplementary Figure 3. Glucose and insulin tolerance of p110α^{DEL} mice over ageing. Cohorts of male (**a**) and female (**b**) p110α^{DEL} and p110α^{FLOX} littermates were subjected to intraperitoneal glucose and insulin tolerance tests at the indicated age points. For glucose tolerance test a bolus of glucose (2 g per kg of body weight) was injected. Insulin doses were adjusted according to the age of the mice and are indicated in each ITT graph. Age and number (n) of mice per genotype (p110α^{FLOX}/p110α^{DEL}) are also indicated.

Data are presented as mean ± SEM. Statistical analysis by unpaired two-tailed t-test. * $p<0.05$; ** $p<0.01$; *** $p<0.001$.

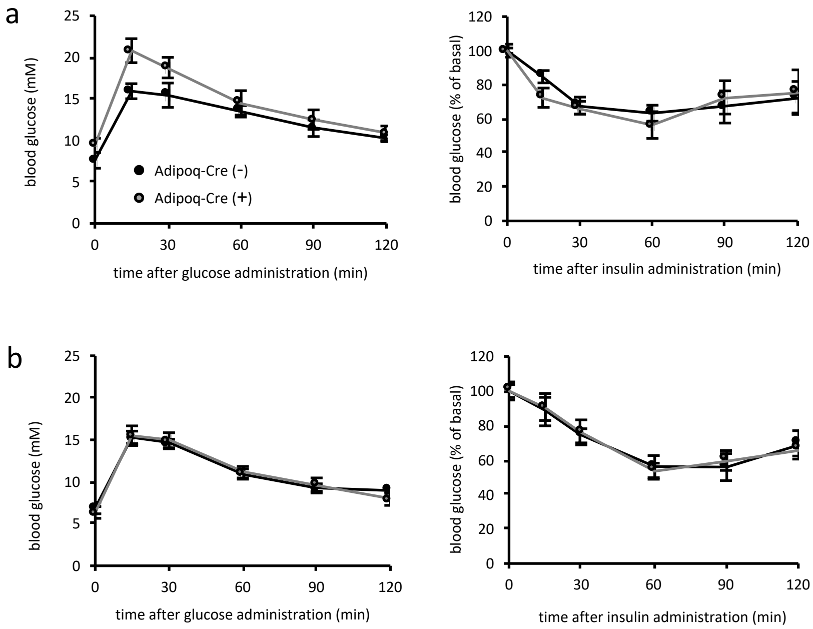

Supplementary Figure 4. Insulin sensitivity and glucose homeostasis are not affected in Adipoq-Cre mice lacking targeted p110α alleles.

Glucose tolerance tests (2 g of glucose/kg of body weight) and insulin tolerance tests (0.75 U of insulin per kg of body weight) were performed in approximately 3 month old males (**a**) (n= 5 Adipoq-Cre(-) / 5 Adipoq-Cre(+)) and females (**b**) (n= 8 Adipoq-Cre(-) / 6 Adipoq-Cre(+)).

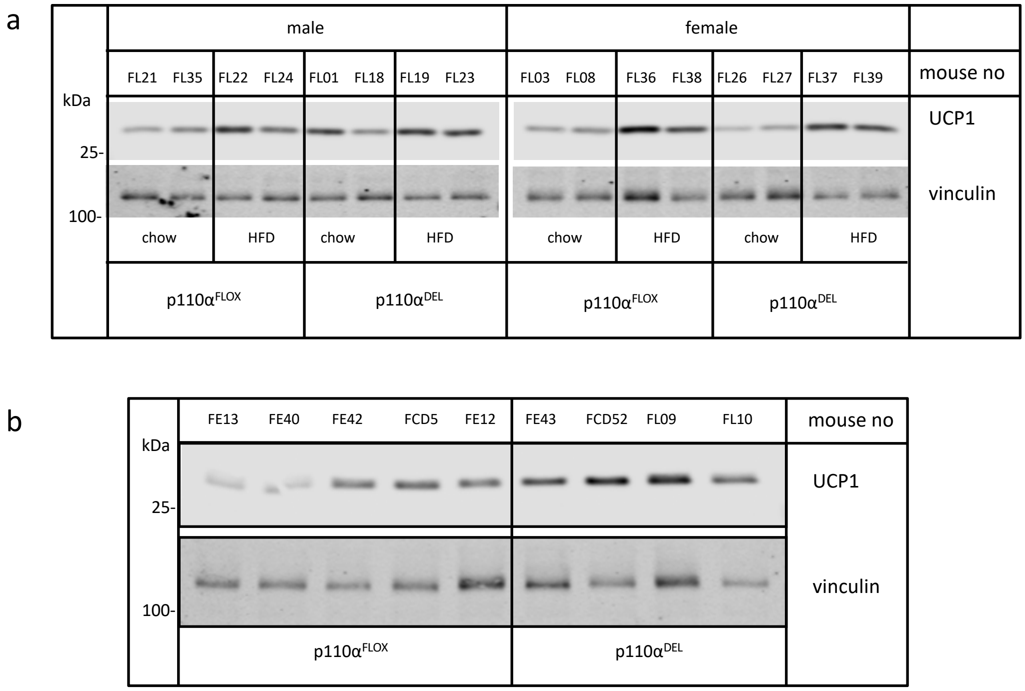

Supplementary Figure 5. Increased UCP1 protein levels in BAT of p110α^{DEL} mice.
a Mice (n=4 per genotype and sex) were fed either standard chow or a high fat diet (HFD, 60% calories from fat) for 4 weeks. Expression of UCP1 was detected by immunoblot analysis in BAT extracts (4 µg of total protein). Vinculin was probed as a loading control. Quantitative data are presented in Fig. 3e.
b Basal levels of UCP1 protein expression detected by immunoblot analysis of BAT extracts (4 µg total protein) of p110α^{FLOX} and p110α^{DEL} mice. Mice are from 8 different litters and 3 different generations. Quantitative data are shown in Fig. 3f. For the calculation of the means shown in Fig. 3f, data of chow fed mice from **a** have been pooled with those of blot in **b**.
UCP1 protein-antibody complexes were detected by infrared imaging using a LICOR Odyssey CLx scanner. Signal intensities were quantified with Image Studio software (LICOR).

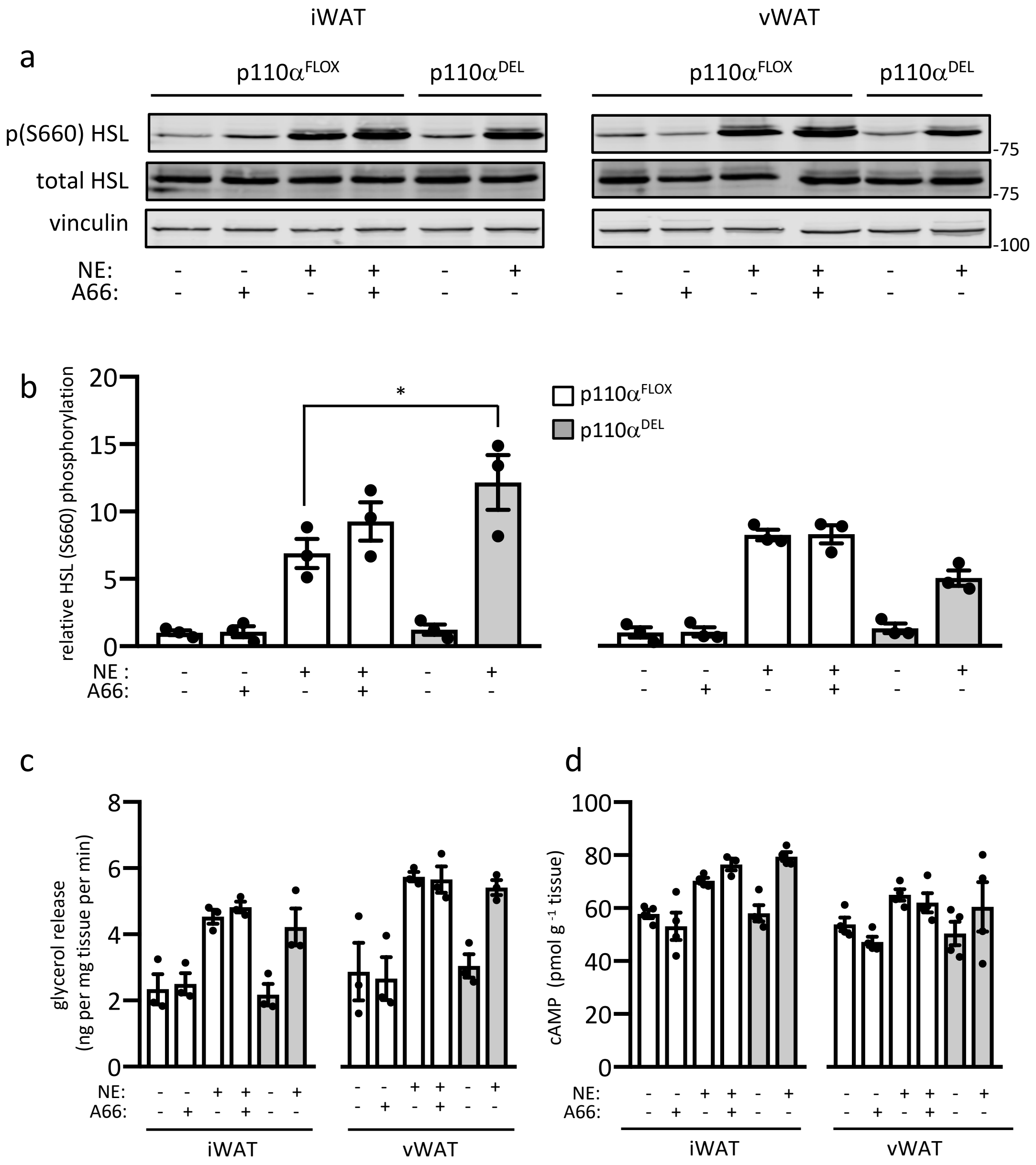

Supplementary Figure 6. Genetic or pharmacological inactivation of p110α potentiates β-adrenergic signalling selectively in mouse iWAT.

a-**b** Explants of inguinal (iWAT) and visceral (vWAT) white adipose tissue were isolated from 1 year old mice and stimulated *ex vivo* with 1 μM Norepinephrine (NE) in the presence or absence of A66 (2 μM) for 20 min, followed by tissue homogenisation and immunoblot analysis of HSL (S660) phosphorylation. A representative immunoblot is shown in **a**. Graphs (**b**) show pooled data from 3 independent experiments.

c Lipolysis in explants from 1 year old male mice (n=3 per genotype) stimulated with 1 μM NE in the presence or absence of 2 μM A66 and assessed as glycerol release over 30 min following stimulation.

d cAMP levels measured by ELISA in explants from approximately 1 year old male mice (n=4 per genotype) stimulated with 1 μM NE in the presence or absence of 2 μM A66 for 20 min.

Data are presented as mean ± SEM. Statistical analysis: One-way ANOVA with Bonferroni's multiple comparisons test. * $p<0.05$.

Araiz *et al.* Supplementary Fig. 7

a

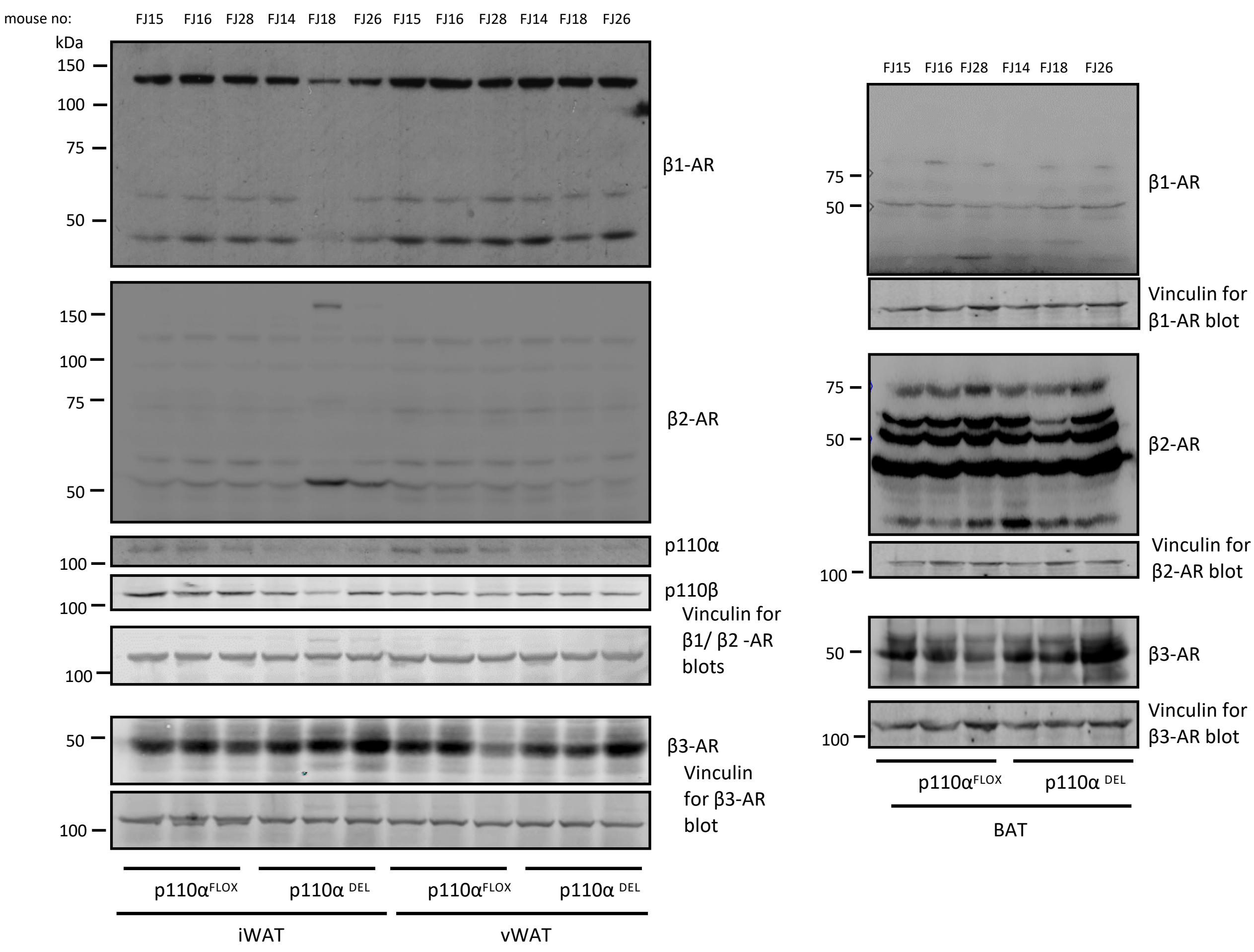

b

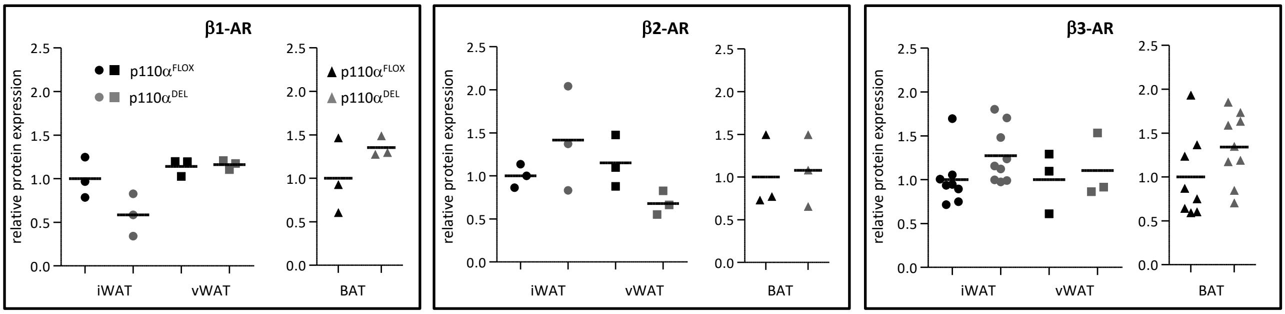

c

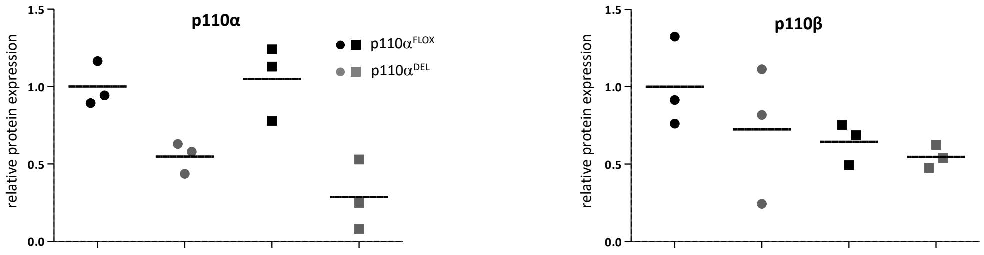

Supplementary Figure 7. β-Adrenergic Receptor protein expression in adipose tissues of p110α^{FLOX} and p110α^{DEL} mice.

a Protein extracts from adipose tissues of 32 week old male p110α^{FLOX} and p110α^{DEL} littermate mice were analysed by immunoblot analysis for adrenergic receptors using the indicated antibodies. β-AR undergo N-glycosylation. The pattern of glycosylation can differ among different cell types. Glycosylation is not thought to affect ligand binding of β-ARs. β1-AR and β2-AR were detected using enhanced chemiluminescence, whereas β3-AR was detected by infrared imaging using a LICOR Odyssey CLx scanner. Signal intensities were quantified with ImageStudio software (LICOR). For β1 and β2-AR expression the whole region visible in the above blot was quantified in each lane. The aminoacid sequence predicted molecular weights for each receptor are : β1-AR 50.5, β2-AR 47, β3-AR 43.5. The membrane used for β2-AR detection was reprobed with antibodies for the p110α and p110β isoforms of PI3K.

b Scatter plots presenting the average intensities of β-ARs and p110α and p110β protein expression in p110α^{FLOX} and p110α^{DEL} mice (n=3 per genotype). β3-AR expression in iWAT and BAT has been probed in lysates from additional mice (in total 8 p110α^{FLOX} and 9 p110α^{DEL} mice derived from 8 different litters and 2 different generations).

c Expression of PI3K p110α and p110β isoforms detected by probing the same blots as in **a** with the isoform-specific antibodies.

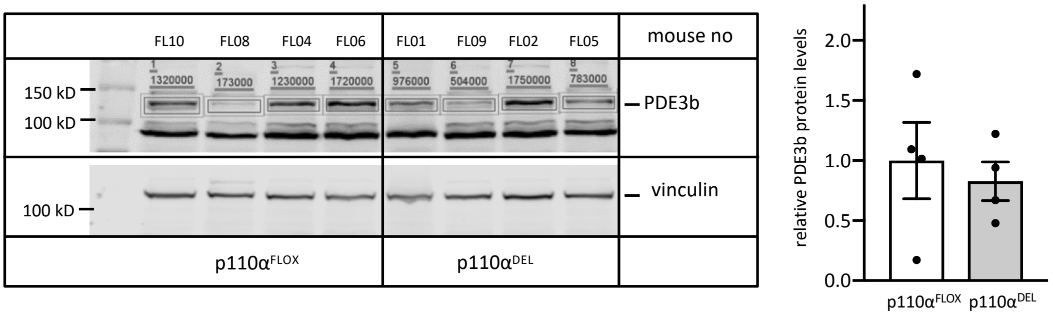

Supplementary Figure 8. Expression of PDE3b in iWAT of p110α^{FLOX} and p110α^{DEL} mice.
PDE3b protein levels detected by immunoblot analysis of inguinal WAT extracts from 10 week old male mice (n=4 per genotype). Vinculin was probed as a loading control. Signals were detected by infrared imaging using a LICOR Odyssey CLx scanner. Signal intensities were quantified with Image Studio software (LICOR).

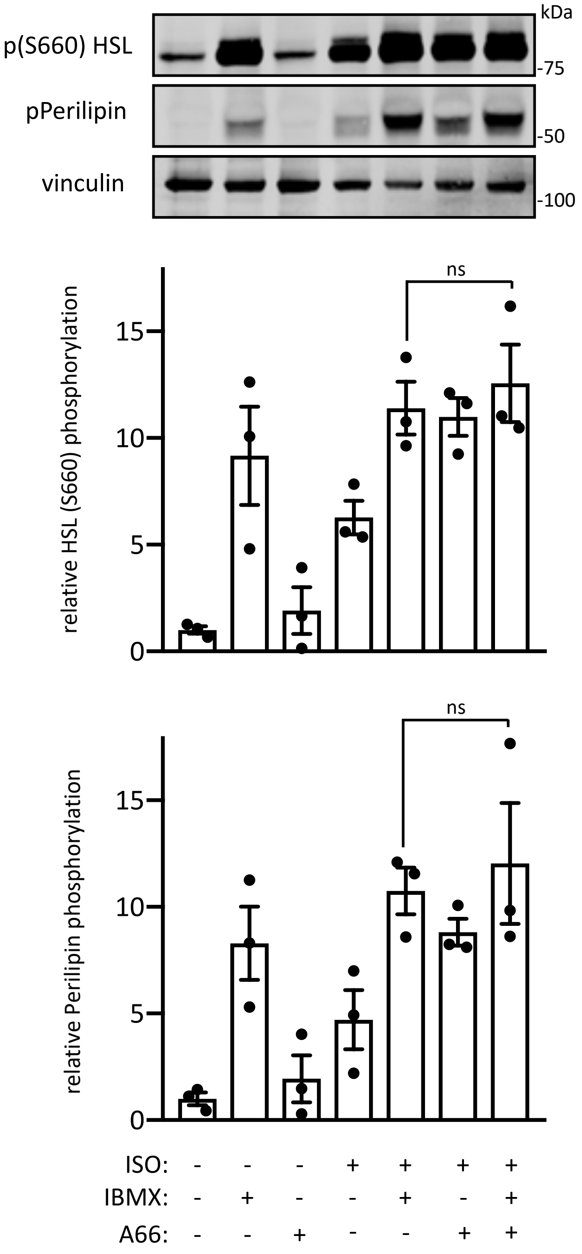

Supplementary Figure 9. Inhibition of p110α does not enhance the stimulatory effect of phosphodiesterase inhibition on adrenergic signalling in inguinal WAT.
Inguinal WAT explants from 38 week old p110α^{FLOX} male mice were stimulated with 1 μM isoproterenol (ISO) in the presence or absence of 2 μM p110α selective inhibitor A66 and/or the broad spectrum phosphodiesterase inhibitor isobutylmethylxanthine (IBMX) at 100 μM for 20 min. Protein extracts were analysed by immunoblot analysis with the indicated antibodies. A66 does not increase the response to ISO stimulation beyond that of treatment with IBMX, consistent with a mechanism of A66 action involving inhibition of phosphodiesterase activity.
Data are presented as mean ± SEM from three independent experiments (n=3). Statistical significance was tested with paired two-tailed t-test.

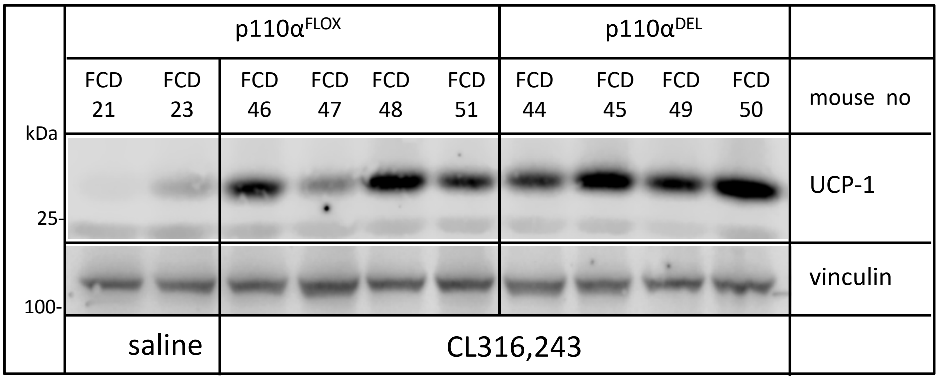

Supplementary Figure 10. UCP1 protein levels in iWAT of p110α^{DEL} mice following β3-AR agonist injections.

a. Male mice (18 week old, n=4 per genotype) were injected intraperitoneally with the β3-AR selective agonist CL316,243 (1 mg per kg per day) over five consecutive days. Expression of UCP-1 was detected by immunoblot analysis in iWAT extracts (100 µg of total protein). Vinculin was probed as a loading control. Quantitative data are presented in Fig. 6.

UCP1 protein-antibody complexes were detected by infrared imaging using a LICOR Odyssey CLx scanner. Signal intensities were quantified with Image Studio software (LICOR).

Fig. 1a immunoblots

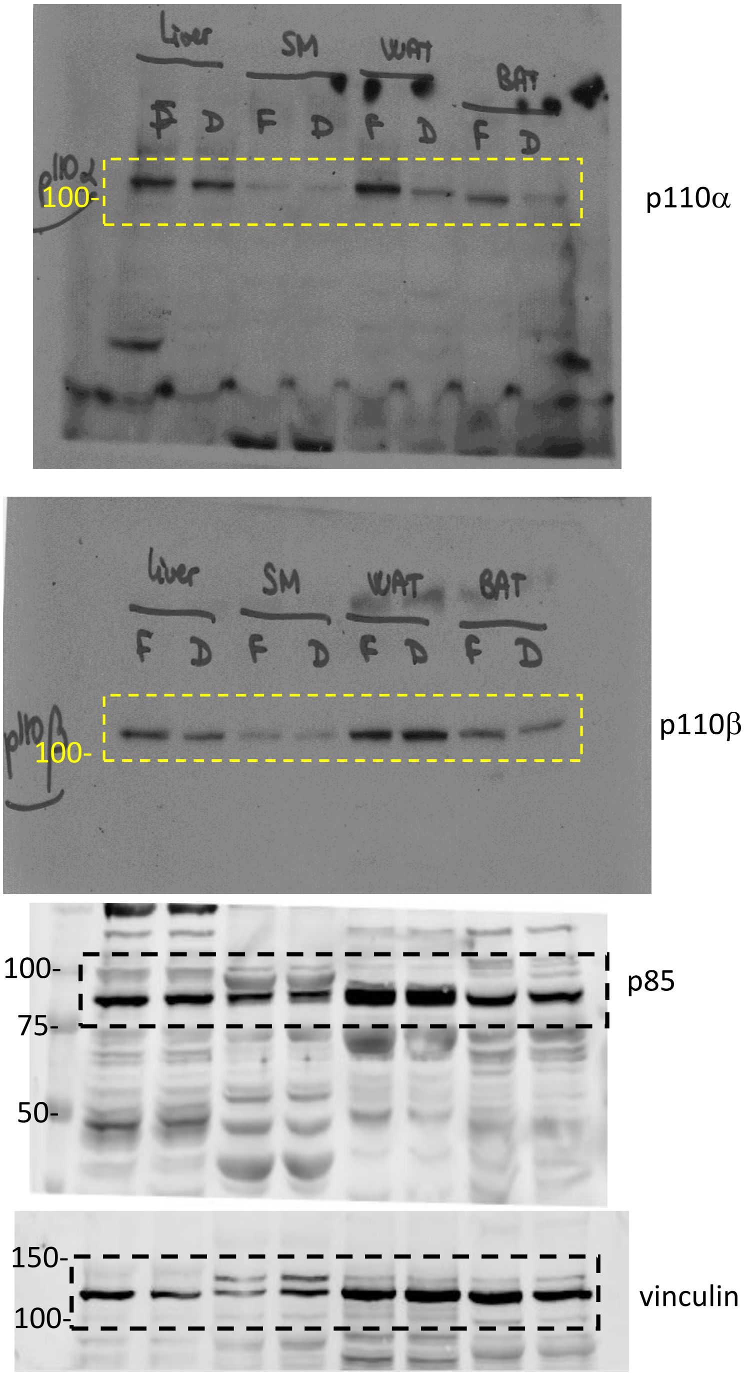

Fig. 1e immunoblot

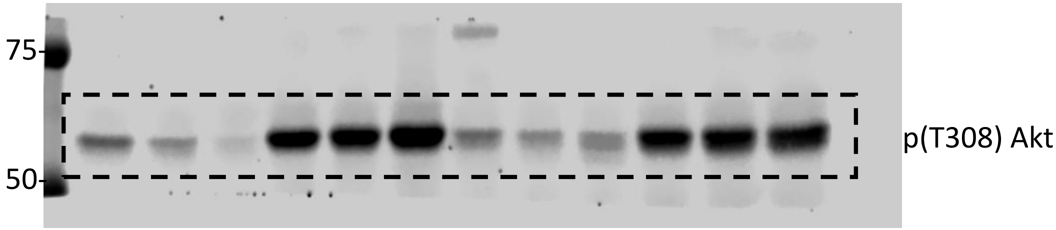

Fig. 4a immunoblots

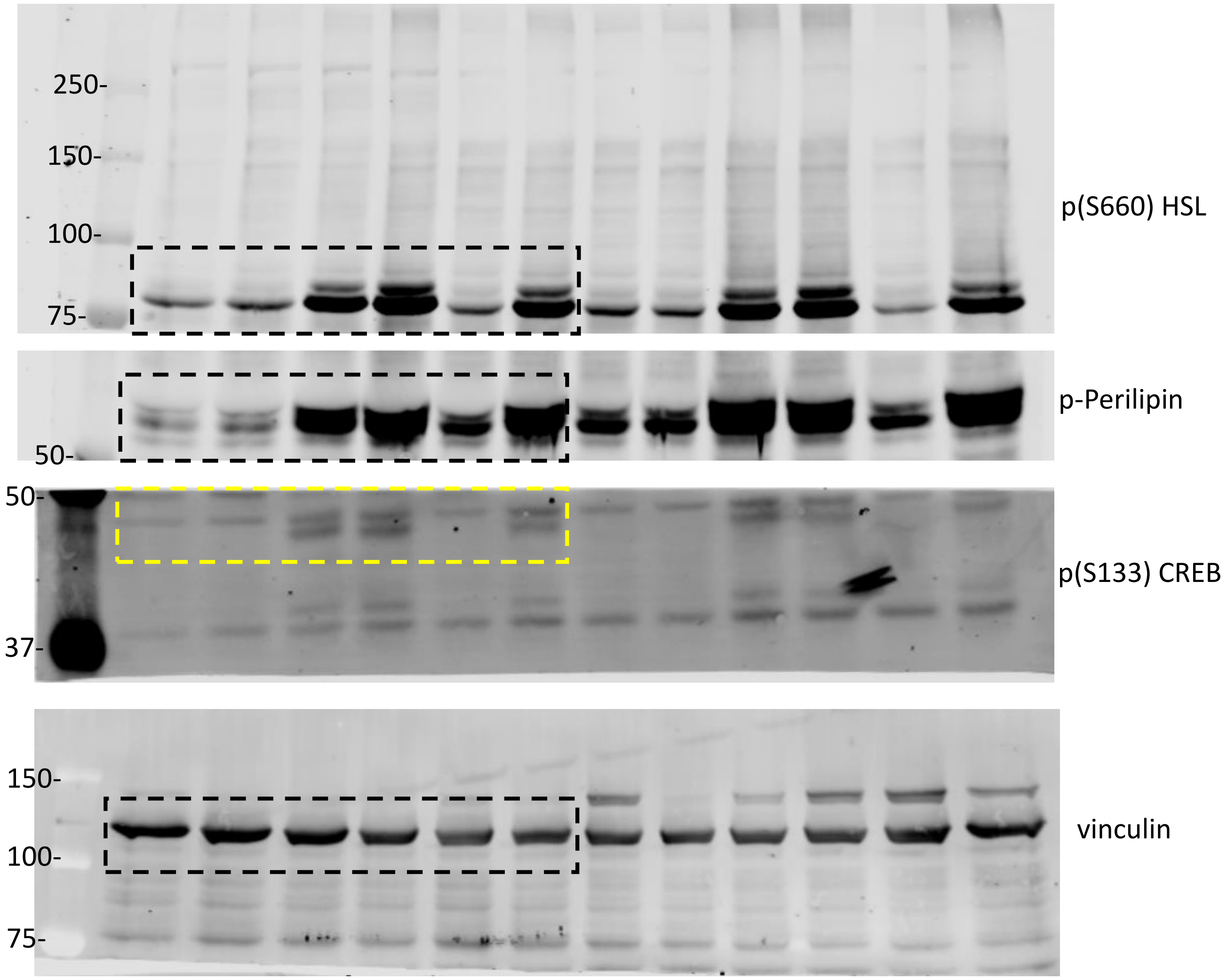

Fig. 5b Immunoblots

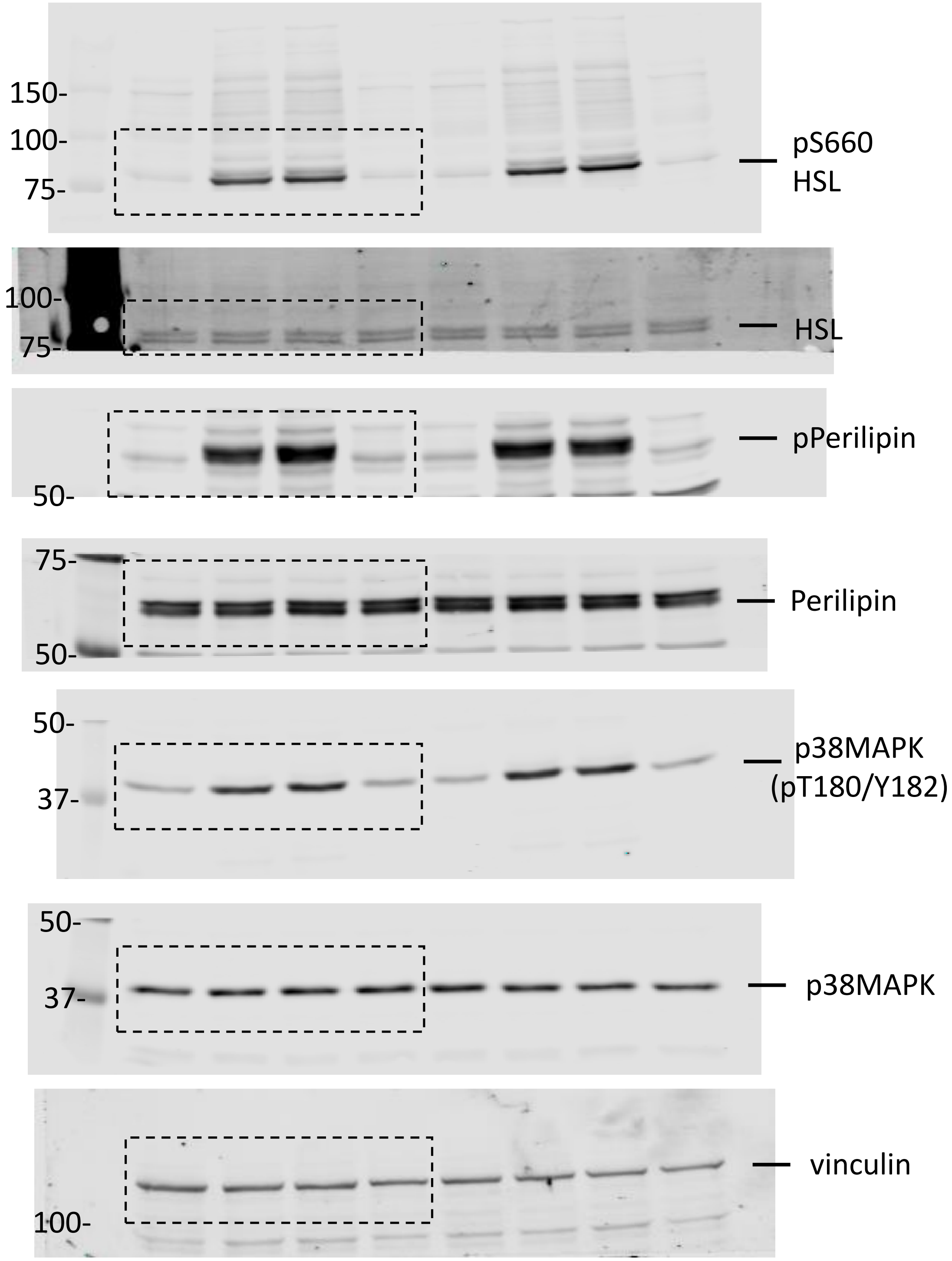

Fig. 6 immunoblot (**iWAT** UCP-1)

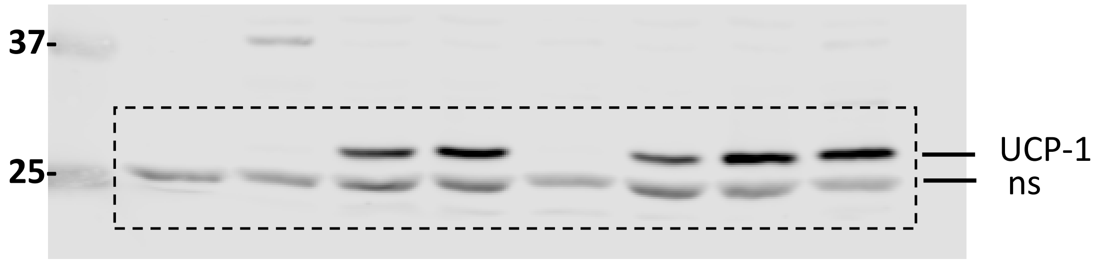

Supplementary Fig. 1 immunoblots

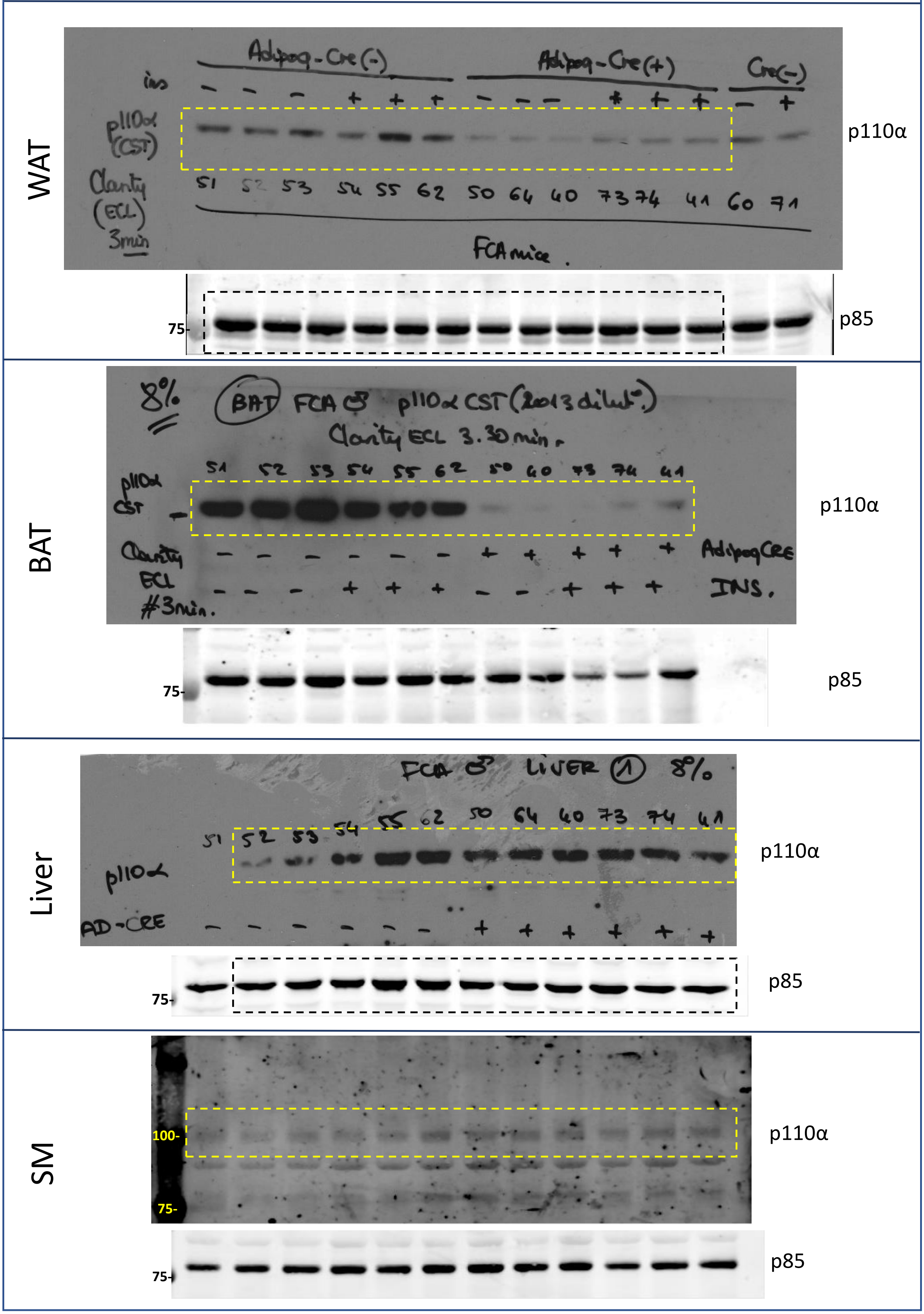

Supplementary Fig. 2 immunoblots

Liver

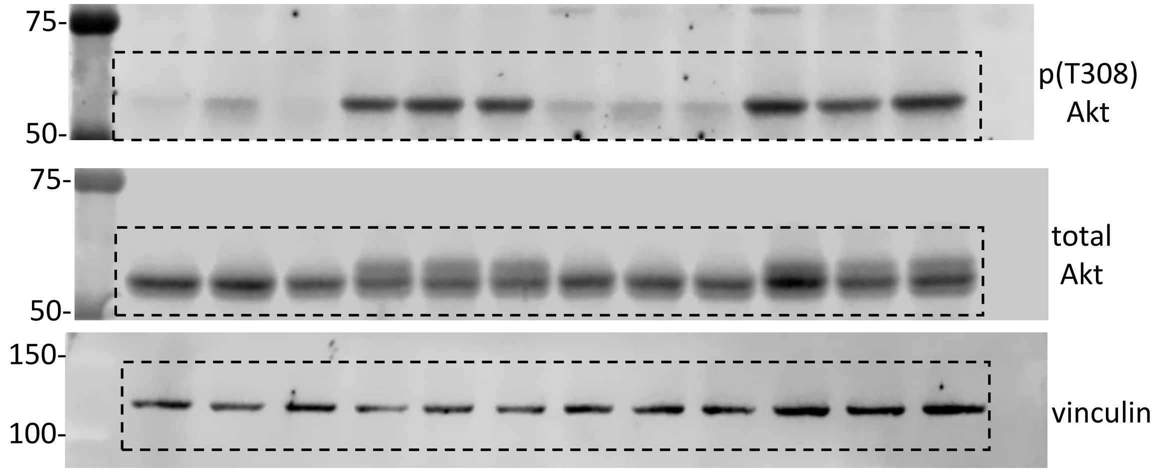

Skeletal Muscle

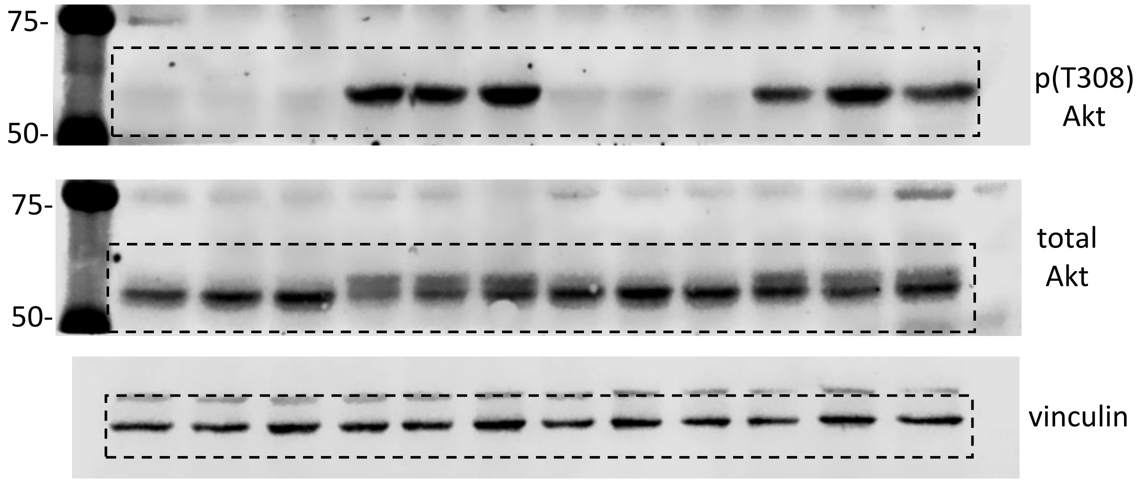

Supplementary Fig. 5 immunoblots (**BAT** UCP-1)

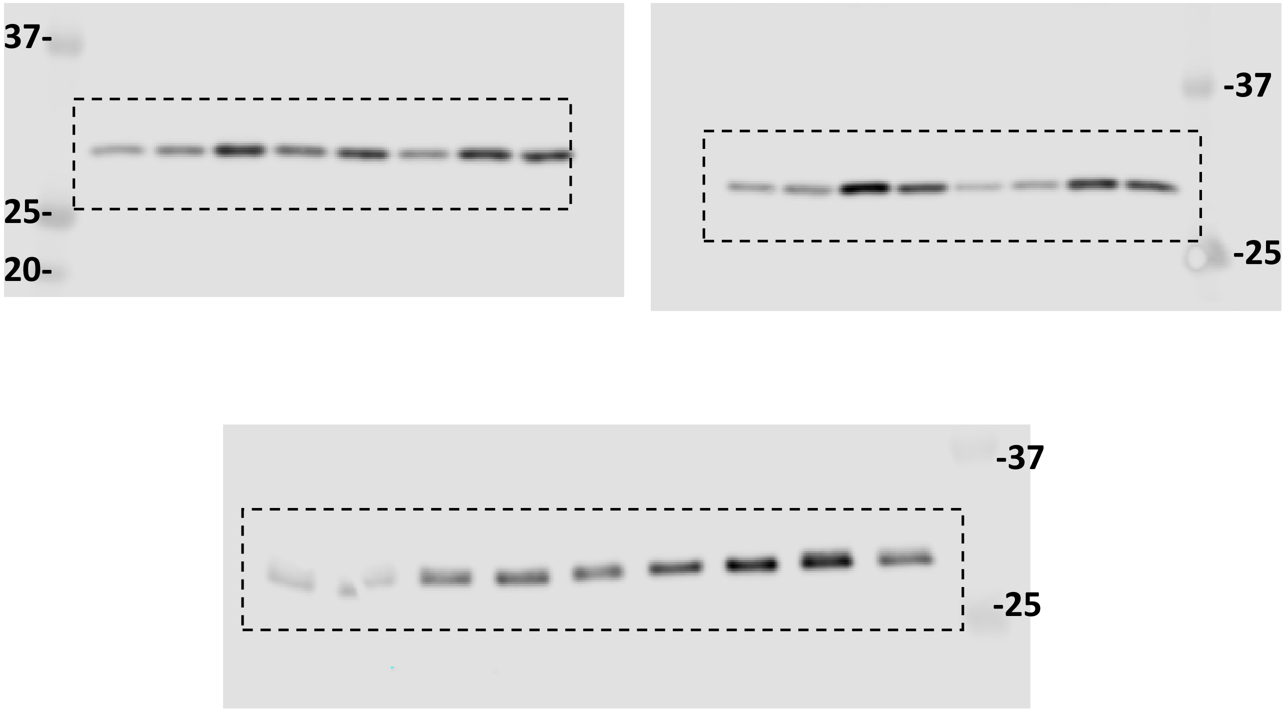

Supplementary Fig. 6 immunoblots

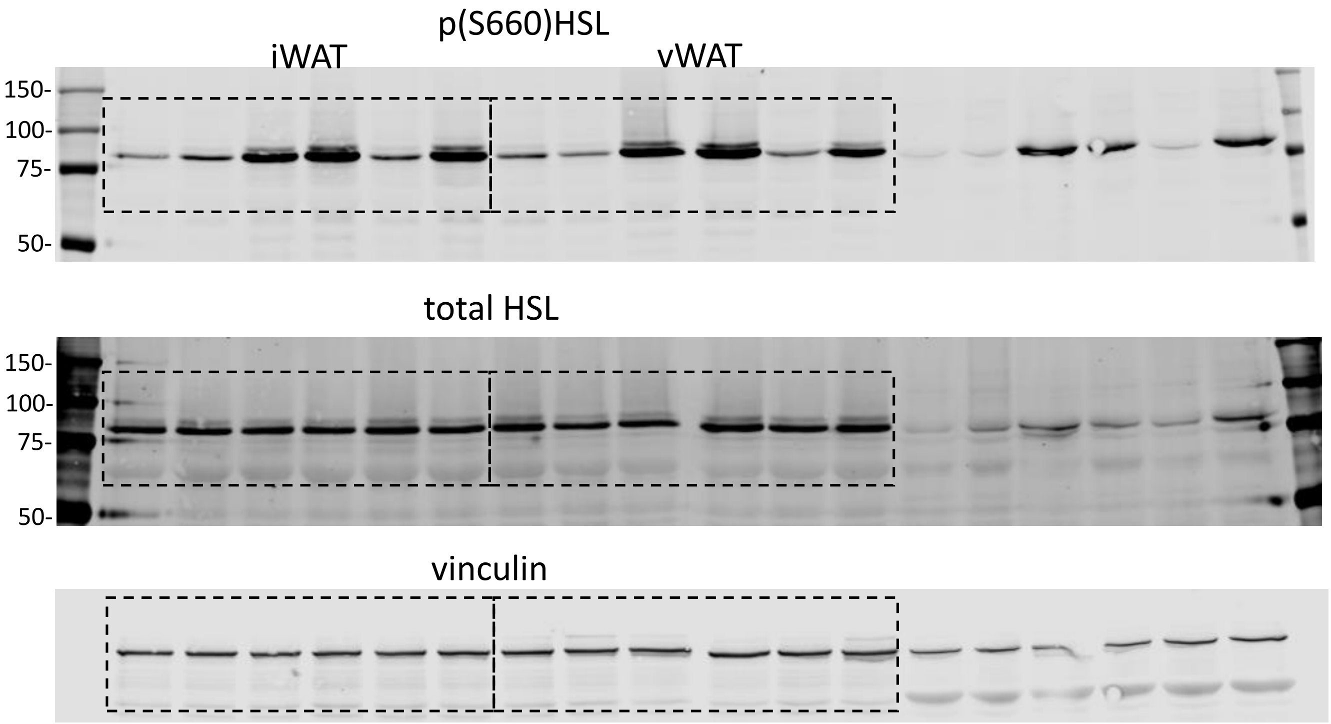

Supplementary Fig. 7 immunoblots

WAT

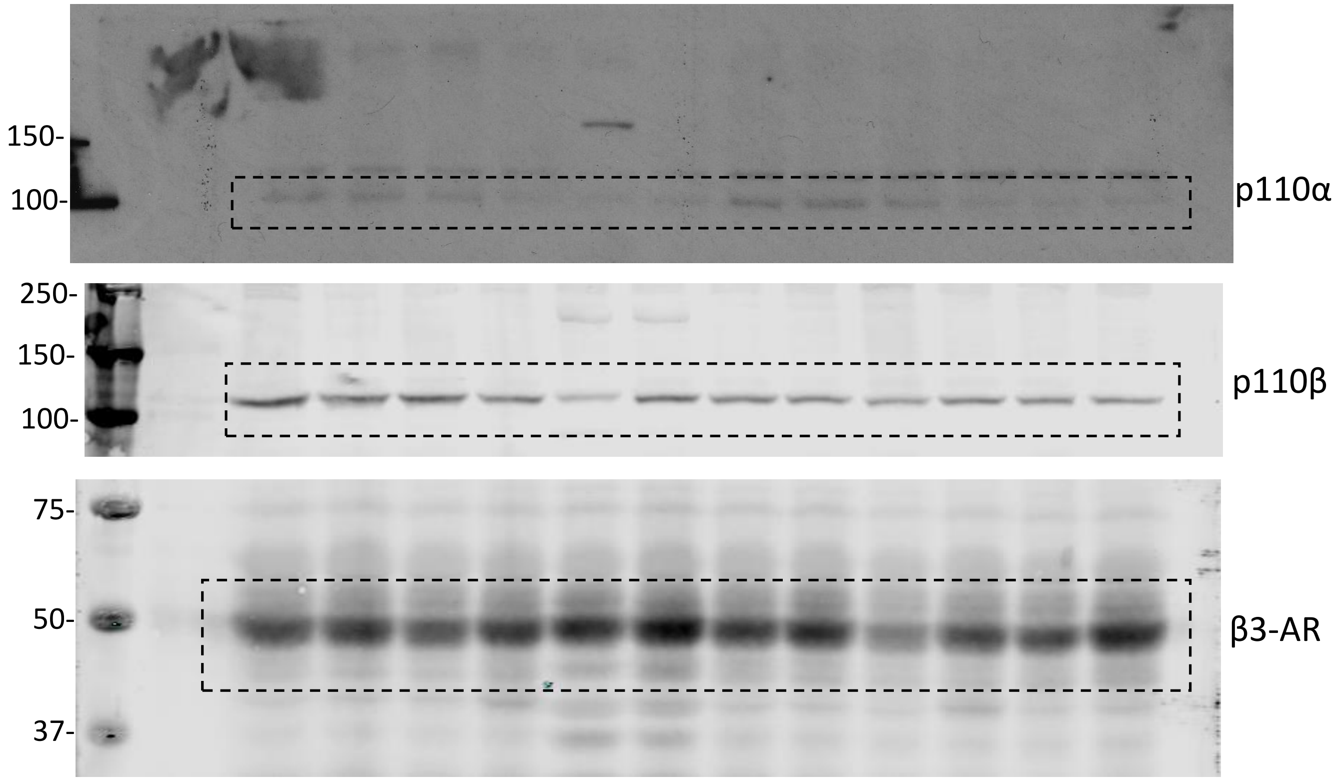

BAT

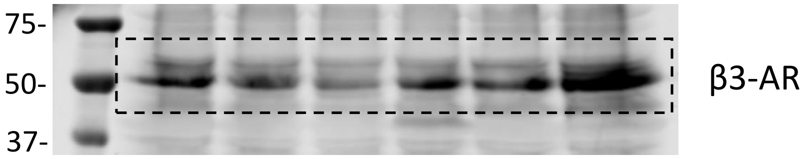

Supplementary Fig. 9 immunoblots

Blot successively probed for pHSL and pPerilipin (without stripping)

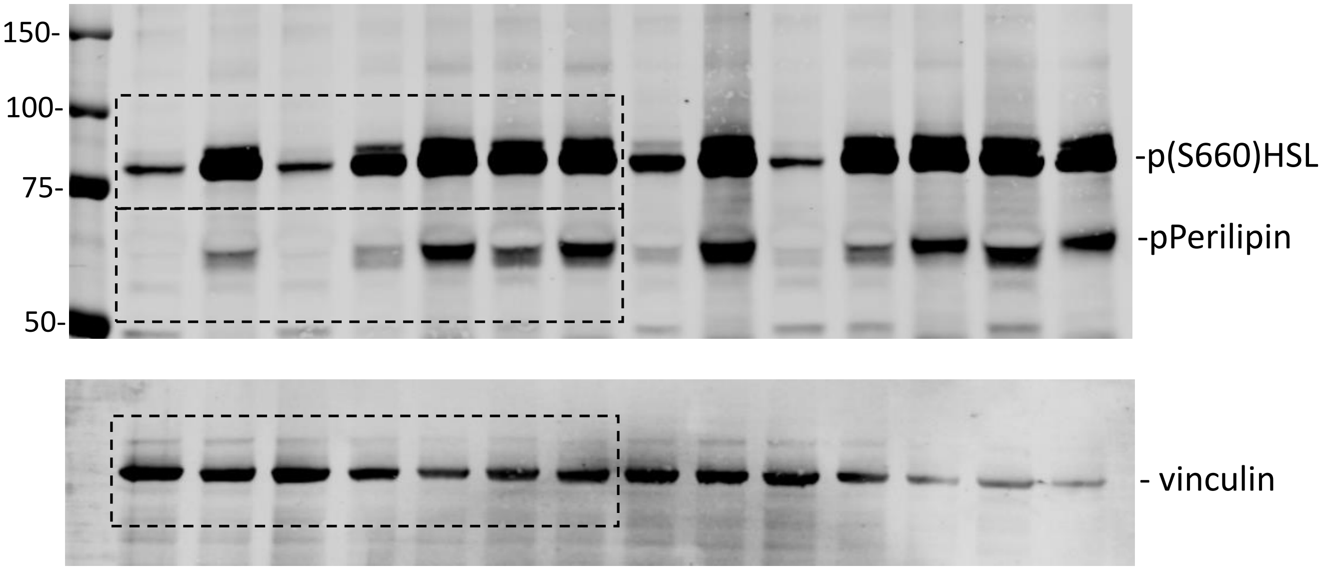

Supplement: Supplementary file 1 — Supplementary Information [file 41467_2019_9514_MOESM1_ESM.pdf]
