# Supplementary material for: Enhanced β-adrenergic signalling underlies an age-dependent beneficial metabolic effect of PI3K p110α inactivation in adipose tissue
Source: Nat Commun. 2019 Apr 4;10:1546. doi: 10.1038/s41467-019-09514-1 (PMC6449391; doi:10.1038/s41467-019-09514-1)
Supplement: Supplementary file 3 — Description of Additional Supplementary Files [file 41467_2019_9514_MOESM3_ESM.pdf]

Description of Supplementary Files

File Name: Supplementary Data 1.

Description: Uncropped images of the most important blots presented in the article.
