# Supplementary material for: Enhanced β-adrenergic signalling underlies an age-dependent beneficial metabolic effect of PI3K p110α inactivation in adipose tissue
Source: Nat Commun. 2019 Apr 4;10:1546. doi: 10.1038/s41467-019-09514-1 (PMC6449391; doi:10.1038/s41467-019-09514-1)
Supplement: Supplementary file 4 — Reporting Summary [file 41467_2019_9514_MOESM4_ESM.pdf]

nature research

Corresponding author(s): Lazaros Foukas

Last updated by author(s): Feb 1, 2019

# Reporting Summary

Nature Research wishes to improve the reproducibility of the work that we publish. This form provides structure for consistency and transparency in reporting. For further information on Nature Research policies, see Authors & Referees and the Editorial Policy Checklist.

## Statistics

For all statistical analyses, confirm that the following items are present in the figure legend, table legend, main text, or Methods section.

| n/a | Confirmed | |
|---|---|---|
| ☐ | ☒ | The exact sample size (*n*) for each experimental group/condition, given as a discrete number and unit of measurement |
| ☐ | ☒ | A statement on whether measurements were taken from distinct samples or whether the same sample was measured repeatedly |
| ☐ | ☒ | The statistical test(s) used AND whether they are one- or two-sided<br>*Only common tests should be described solely by name; describe more complex techniques in the Methods section.* |
| ☒ | ☐ | A description of all covariates tested |
| ☐ | ☒ | A description of any assumptions or corrections, such as tests of normality and adjustment for multiple comparisons |
| ☐ | ☒ | A full description of the statistical parameters including central tendency (e.g. means) or other basic estimates (e.g. regression coefficient) AND variation (e.g. standard deviation) or associated estimates of uncertainty (e.g. confidence intervals) |
| ☐ | ☒ | For null hypothesis testing, the test statistic (e.g. *F*, *t*, *r*) with confidence intervals, effect sizes, degrees of freedom and *P* value noted<br>*Give P values as exact values whenever suitable.* |
| ☒ | ☐ | For Bayesian analysis, information on the choice of priors and Markov chain Monte Carlo settings |
| ☒ | ☐ | For hierarchical and complex designs, identification of the appropriate level for tests and full reporting of outcomes |
| ☒ | ☐ | Estimates of effect sizes (e.g. Cohen's *d*, Pearson's *r*), indicating how they were calculated |

*Our web collection on statistics for biologists contains articles on many of the points above.*

## Software and code

Policy information about availability of computer code

| | |
|---|---|
| Data collection | no software was used |
| Data analysis | ImageJ , ImageStudio Light 5.2 (LICOR) |

For manuscripts utilizing custom algorithms or software that are central to the research but not yet described in published literature, software must be made available to editors/reviewers. We strongly encourage code deposition in a community repository (e.g. GitHub). See the Nature Research guidelines for submitting code & software for further information.

## Data

Policy information about availability of data

All manuscripts must include a data availability statement. This statement should provide the following information, where applicable:

- Accession codes, unique identifiers, or web links for publicly available datasets
- A list of figures that have associated raw data
- A description of any restrictions on data availability

The data that support the findings of this study are included in this published article and its Supplementary Information files. All other relevant data are available from the corresponding author upon reasonable request.

# Field-specific reporting

Please select the one below that is the best fit for your research. If you are not sure, read the appropriate sections before making your selection.

☒ Life sciences ☐ Behavioural & social sciences ☐ Ecological, evolutionary & environmental sciences

For a reference copy of the document with all sections, see nature.com/documents/nr-reporting-summary-flat.pdf

# Life sciences study design

All studies must disclose on these points even when the disclosure is negative.

| | |
|---|---|
| Sample size | Sample sizes for animal experiments were decided based on previous experience with the various assays or on results of pilot experiments. No statistical methods were used to predetermine the sample size. |
| Data exclusions | No data were excluded (except in rare occasions of inability to obtain a measurement due to technical failure). |
| Replication | Most animal metabolic experiments have been repeated with independent cohorts from different generations and most of the reported phenotypes have been confirmed over various age points of the same cohorts as this has been a longitudinal (ageing) study. Data from cells or tissue explants have been repeated at least in three independent occasions. |
| Randomization | Animals were randomized to experimental groups. |
| Blinding | N/A. No assays used requiring data scoring by an operator. Data values were calculated by computer software. |

# Reporting for specific materials, systems and methods

We require information from authors about some types of materials, experimental systems and methods used in many studies. Here, indicate whether each material, system or method listed is relevant to your study. If you are not sure if a list item applies to your research, read the appropriate section before selecting a response.

## Materials & experimental systems

| n/a | Involved in the study | |
|---|---|---|
| ☐ | ☒ | Antibodies |
| ☐ | ☒ | Eukaryotic cell lines |
| ☒ | ☐ | Palaeontology |
| ☐ | ☒ | Animals and other organisms |
| ☒ | ☐ | Human research participants |
| ☒ | ☐ | Clinical data |

## Methods

| n/a | Involved in the study | |
|---|---|---|
| ☒ | ☐ | ChIP-seq |
| ☒ | ☐ | Flow cytometry |
| ☒ | ☐ | MRI-based neuroimaging |

## Antibodies

| | |
|---|---|
| Antibodies used | p110α (Cell Signaling Technology (CST), cat. no. 4255, diluted 1:1,000), p110β (CST, cat. no. 3011, diluted 1:1,000), pT308 Akt (CST, cat. no. 2965, diluted 1:1,000), pS473 (CST, cat. no. 4060, diluted 1:1,000), total Akt (CST, cat. no. 9272, diluted 1:1,000), pS660 HSL (CST, cat. no. 4126, diluted 1:1,000), total HSL (CST, cat. no. 4107, diluted 1:1,000), phosphoPKA substrate (CST, cat. no. 9624, diluted 1:1,000), total perilipin (CST, cat. no. 9349, diluted 1:1,000), pS133 CREB (CST, cat. no. 9191, diluted 1:1,000), pT180/Y182 p38 MAPK (CST, cat. no. 9215, diluted 1:1,000), total p38 MAPK (CST, cat. no 9212, diluted 1:1,000). UCP1 antibody was from Abcam (cat. no. 10983, diluted 1:1,000), p85 (Millipore, cat. no. 06-195, diluted 1:1,000), IRS-1 (Millipore, cat.no 05-1085), PDE3b (StJohn's Laboratory, cat. no. STJ110746, diluted 1:1,000), Vinculin (Sigma, cat. no. V9264, diluted 1: 10,000). |
| Validation | All antibodies used were of commercial origin and have been validated by the respective manufacturers. |

## Eukaryotic cell lines

Policy information about cell lines

| | |
|---|---|
| Cell line source(s) | hMADS were provided by the proprietor. |
| Authentication | hMADS are not immortalised. An early passage of hMADS was provided by the establishing lab. |
| Mycoplasma contamination | Free of mycoplasma contamination. |
| Commonly misidentified lines (See ICLAC register) | n/a |

## Animals and other organisms

Policy information about studies involving animals; ARRIVE guidelines recommended for reporting animal research

| | |
|---|---|
| Laboratory animals | Mice, C57/BL6J of both sexes and various ages. |

| | |
|---|---|
| Wild animals | n/a |
| Field-collected samples | n/a |
| Ethics oversight | All experimental procedures complied with the UK Home Office Animals (Scientific Procedures) Act 1986 and were performed with the approval of the UCL Animal Welfare and Ethical Review Body. |

Note that full information on the approval of the study protocol must also be provided in the manuscript.
